# Male Ejaculatory Endophenotypes: Revealing Internal Inconsistencies of the Concept in Heterosexual Copulating Rats

**DOI:** 10.3389/fnbeh.2020.00090

**Published:** 2020-06-26

**Authors:** Itztli Trejo-Sánchez, Carlos Pérez-Monter, Sofía Huerta-Pacheco, Gabriel Gutiérrez-Ospina

**Affiliations:** ^1^Laboratorio de Biología de Sistemas, Instituto de Investigaciones Biomédicas, Departamento de Biología Celular y Fisiología, Universidad Nacional Autónoma de México, Mexico City, Mexico; ^2^Programa de Doctorado en Ciencias Biomédicas, Unidad de Posgrado, Universidad Nacional Autónoma de México, Mexico City, Mexico; ^3^Departamento de Gastroenterología, Instituto Nacional de Ciencias Médicas y Nutrición “Salvador Zubirán”, Mexico City, Mexico; ^4^Consejo Nacional de Ciencia y Tecnología, Ciencia Forense, Facultad de Medicina, Universidad Nacional Autónoma de México, Mexico City, Mexico; ^5^Coordinación de Psicobiología y Neurociencias, Facultad de Psicología, Universidad Nacional Autónoma de México, Mexico City, Mexico

**Keywords:** sexual diversity, sexual phenotypes, sexual brain, male copulatory behavior, sexual continuum, sexual fluidity, brain mosaic, sexual behavior

## Abstract

Distinct manifestations of sexual behavior are conceived as separate phenotypes. Each sexual phenotype is assumed to be associated with a characteristic brain. These notions have justified the phenotyping of heterosexual copulator males based upon their ejaculation’s latencies (EL) or frequencies (i.e., cumulative ejaculation number; EN). For instance, men and male rats showing premature, normal or retarded ejaculation are assumed to be distinctive endophenotypes. This concept, nonetheless, contradicts past and recent evidence that supports that sexual behavior is highly variable within each sex, and that the brain sexual functional morphology represents an intricate sexual phenotypic mosaic. Hence, for ejaculatory male endophenotypes to be considered as a valid biological concept, it must show internal consistency at various levels of organization (including genetic architectures), after being challenged by intrinsic and/or extrinsic factors. We then judged the internal consistency of the presumed ejaculatory endophenotypes by assessing whether copulatory behavior and the expression of copulation relevant genes and brain limbic structures are specific to each of the presumed EL- or EN-ejaculatory endophenotypes. To do this, copulating male rats were first phenotyped in groups consistently displaying short, average or long ejaculation latencies or very high, high, average, low or very low EN, based in their copulatory performance. Then, the internal consistency of the presumed EL- or EN-endophenotypes was tested by introducing as covariates of phenotyping other copulatory parameters (e.g., number of intromissions) in addition to EL or EN, or by analyzing the expression levels of genes encoding for estrogen receptor alpha, progesterone receptor, androgen receptor, aromatase, DNA methyl-transferase 3a and DNA methyl-transferase 1 in the amygdala, medial preoptic area, ventromedial hypothalamus and olfactory bulb. We found that even though there were group-level differences in all the variables that were studied, these differences did not add-up to create the presumed EL- or EN-ejaculatory endophenotypes. In fact, the extensive overlapping of copulatory parameters and expression levels of copulation relevant genes in limbic structures across EL- or EN-phenotyped copulating male rats, is not consistent with the hypothesis that distinct ejaculatory endophenotypes exist and that they are associated with specific brain characteristics.

## Introduction

Distinct manifestations of sexual behavior (e.g., female/male heterosexuality, male/female homosexuality; female/male bisexuality and so forth) are long thought to represent separate phenotypes (Chivers et al., 2004; de Vries and Södersten, 2009; Jordan, 2010; Cerny and Janssen, 2011; Flanagan-Cato, 2011; Rosenthal et al., 2011, 2012; Balthazart, 2016; Joel and Fausto-Sterling, 2016; Portillo and Paredes, 2019). This notion not only embraces the behavioral display of the individuals sharing distinct sexual expressions, but also the presumption that the brain of each phenotypic group displays functional morphological attributes that are specific to each sexual phenotype (Zhou et al., 1995; Fernández-Guasti et al., 2000; Kruijver et al., 2000; Savic et al., 2005, 2010; Berglund et al., 2006; Swaab, 2008; Sakamoto, 2012; Rahman and Yusuf, 2015; Taziaux et al., 2016; Burke et al., 2017; Amezcua-Gutiérrez et al., 2018). Accordingly, in humans, it has been reported that (1) the functional organization of the brain of heterosexual women and men differs importantly from each other (Savic and Lindstrom, 2008), (2) that the brain’s functional organization of homosexual men and women differs one another with the former laying closer to the brain’s organization reported for heterosexual females, and the latter nearer to that reported for heterosexual males (Savic and Lindstrom, 2008), and (3) that the brain’s functional organization of bisexual individuals charts somewhere in between the brain organization reported for monosexual, hetero- or homo-sexual men (Safron et al., 2017). A similar scenario has been drawn for hypersexual (Absher, 2016), asexual (Prause and Harenski, 2014) and transsexual (Garcia-Falgueras and Swaab, 2008) individuals. At least some of these phenotypes are presumed to result from specific genetic architectures (e.g., Arnold and Chen, 2009; Ngun et al., 2011; Arnold, 2017), hormonal (e.g., Wilson et al., 1981; de Vries and Södersten, 2009; Hines, 2010; Olvera-Hernández and Fernández-Guasti, 2015) and epigenetic (Rice et al., 2013; McCarthy and Nugent, 2015; Nugent et al., 2015, 2017; Forger, 2016; Mosley et al., 2017; McCarthy, 2019) makeups, so they may be considered as endophenotypes (e.g., Rahman, 2005; Ponseti et al., 2006).

The construct that claims the existence of distinct sexual phenotypes is not applied only to human beings, but also to other animal species (for a comprehensive review see de Vries and Södersten, 2009; also Jazin and Cahill, 2010; Forger, 2016; Ventura-Aquino and Paredes, 2017; LaClair et al., 2019). In this regard, female- or male-preferring male rats (Coria-Avila, 2012; Coria-Avila et al., 2014; Olvera-Hernández and Fernández-Guasti, 2015) and rams (Alexander et al., 1993, 1999; Borja and Fabre-Nys, 2012; Sutton et al., 2018), as well as copulating and non-copulating male rats (Portillo and Paredes, 2003, 2019; Portillo et al., 2006a, b; Ventura-Aquino and Paredes, 2017) are good examples to keep in mind because they are thought to represent distinct copulatory phenotypes associated each to a characteristic brain. Accordingly, previous studies sustain that the expression levels of sexually relevant genes such as those encoding estrogen, progesterone and androgen receptors and the enzyme aromatase in the amygdala, medial preoptic area and the olfactory bulb, distinctively differ between non-copulating and copulating male rats (Portillo et al., 2006a, b; Antaramian et al., 2015), even though serum testosterone levels does not differ between them (Portillo et al., 2010). This last observation is not surprising since it has been long known that under physiological conditions, in male rats, ejaculation is minimally affected by testosterone (Whalen et al., 1961), there is a limited correlation between circulating testosterone and sexual behavior (Damassa et al., 1977), chronic sexual activity does not predict testosterone concentration (Shulman and Spritzer, 2014; see also Portillo et al., 2010) and there is no association between ejaculation times and testosterone levels (Morgentaler et al., 2017).

In relatively recent times, the idea that males, at least in mammals, display distinct ejaculatory endophenotypes has been proposed. Indeed, it is said, for instance, that men and male rats showing premature, normal or retarded ejaculation represent distinctive endophenotypes (Pattij et al., 2005; Waldinger and Olivier, 2005; Olivier et al., 2006; Ventura-Aquino and Paredes, 2017). These claims come across in spite of the long-held recognition that, with regard to sexual traits, intrasexual variability is as large as intersexual variability (Whalen, 1991), that the origin of brain sexual differences within and across sexual populations is multifaceted (McCarthy et al., 2018) and that there might be a continuum of sexual traits (Epstein et al., 2012; Walton et al., 2016) that could explain sexual fluidity (Diamond, 2016; Diamond et al., 2017; Luoto et al., 2019) within and across the sexes. In addition, ejaculatory endophenotypes, as it is the case for other endophenotypes (e.g., Gottesman and Gould, 2003; Burmeister et al., 2008; Iacono et al., 2014; Jonas and Markon, 2014; for critical reviews see Flint and Munafò, 2007; Walters and Owen, 2007), are even alleged to be genetically determined (Pattij et al., 2005; Waldinger and Olivier, 2005; Olivier et al., 2006; Santtila et al., 2010; Abdel-Hamid and Ali, 2018; Olivier and Olivier, 2019), and therefore they must be the heritable, state-independent, and largely immune to social context and sexual experience once the ejaculatory pattern has been established. In addition, ejaculatory endophenotypes might also be associated to characteristic brains (Ozcan et al., 2001; Waldinger and Schweitzer, 2005; Hyun et al., 2008; Chen, 2016; Zhang et al., 2017; Yang et al., 2018). This last view is also at odds with data showing that (1) individual brains are sexually mutable, mosaic-type variations of a common brain design (Joel, 2011, 2012; Joel et al., 2015; Joel and Fausto-Sterling, 2016), (2) there exists an intersexual nature of neuroendocrinological, behavioral and psychosocial traits at least between males and females (de Vries and Södersten, 2009; Crews, 2012; Joel, 2012; Joel and Yankelevitch-Yahav, 2014; Hyde et al., 2019), (3) maternally and paternally, mono- or biallelic genomic imprinted cells populate and continuously adjust their gene expression patterns following a mosaic, intersexed pattern (Keverne, 2013; Ho-Shing and Dulac, 2019), (4) functional neuronal circuits underlying male- or female-specific behavior coexist in normal female or male mouse brains (de Vries and Södersten, 2009), and (5) genes may not allow meaningful prediction of individual sexual preference (Ganna et al., 2019). Clearly, the internal consistency of the presumption supporting the existence of male ejaculatory endophenotypes must be independently confirmed.

We then assumed this task by using heterosexual, copulator male rats phenotyped based upon their ejaculation latency (EL) or number (EN), after being tested in copulatory contests (e.g., Pattij et al., 2005). Then, the internal consistency of the presumed ejaculatory phenotypes was assessed by introducing as covariates of phenotyping other copulatory parameters in addition to EL or EF and the expression levels of estrogen receptor alpha (ESR1), progesterone receptor (PR), androgen receptor (AR), aromatase (CPY19), DNA methyl-transferase 3a (DNMT3a) and DNA methyl-transferase 1 (DNMT1) in the amygdala (AMG), medial preoptic area (MPOA), ventromedial hypothalamus (VMH) and olfactory bulb (OB), all gene products and brain areas involved in the regulation of male copulation and ejaculation (Holstege et al., 2003; Hull and Dominguez, 2007; Hull and Rodríguez-Manzo, 2009); these genes and brain regions are particularly important since ejaculation as a potential endophenotypic trait, is a subprocess of copulation. So, if ejaculatory endophenotypes were internally consistent, both copulatory behavior and the expression patterns of sexually relevant genes in the limbic structures evaluated should be reasonably specific to each of the presumed, male ejaculatory endophenotypes. We found that even though there were group-level differences in all the variables that were studied, these differences did not add-up to create the presumed, EL- or EN-ejaculatory endophenotypes. Hence, the overlapping of the copulatory parameters and expression levels of copulation relevant genes in limbic structures across the EL- or EN-phenotyped male rats, reveals an intrinsic inconsistency of the concept that presumes the existence of ejaculatory endophenotypes in male rats. Contrary to the prediction, all copulating male rats seem to have sexual behavior displays and brain phenotypes shared by most of the presumed ejaculatory endophenotypes.

## Materials and Methods

### Animals

Sexually naïve male (250–300 g; *n* = 50) and female (200–250 g; *n* = 50) Wistar rats were provided by the colony sheltered by the Unidad de Modelos Biológicos at the Insituto de Investigaciones Biomédicas (IIB), Universidad Nacional Autónoma de México (UNAM). After 3 days of handling, randomly selected animals were housed in groups of five individuals per cage and kept in a room at 25°C under inverted 12/12 h, light-dark cycles (lights off at 7:00 AM). At all times, rats had free access to standard rat chow and water. Female rats were bilaterally ovariectomized under general anesthesia. After full recovery (1 week later), they were primed, for 1 week, with estradiol benzoate injected subcutaneously (25 μg; Sigma-Aldrich, St. Louis, MO, United States) every 48 h. The primed females were rendered sexually receptive by administering to them progesterone (1 mg; Sigma-Aldrich), 3–4 h before conducting the copulatory tests. Animal handling and experimental procedures followed the Mexican official norm NOM-062-ZOO-1999 “Especificaciones técnicas para la producción, cuidado y uso de los animales del laboratorio.” In addition, all the procedures were approved by the Comisión Institucional para el Cuidado y Uso de Animales del Laboratorio (Permit No. 163) at IIB, UNAM.

### Copulatory Tests and Male Phenotypin*g*

Six monandrous copulatory tests, one per week, were scheduled for each sexually naïve copulating male. Under this training scheme, male rats commonly achieve consistent copulatory performance after four successful copulatory encounters (i.e., achieve ejaculation) with receptive females (Pattij et al., 2005; Olivier and Olivier, 2019); consistent copulatory performance changes little over time (Olivier et al., 2006; Olivier and Olivier, 2019), even under conditions that expose males to increasing sexual experience (Thonhauser et al., 2019). All tests (each lasting 30 min; Pattij et al., 2005) were conducted under red dim light, during the dark phase of the day/night cycle. As each session progressed, the observer recorded ejaculation (EL), mount (ML), intromission (IL) latencies and numbers. Then, EL, ML, and IL means per animal were estimated based on the values obtained in every ejaculatory series along the six copulatory tests. Latency values represent the number of seconds that elapses from the introduction of the female into the copulatory arena until the moment in which the male executes the first mount, intromission or ejaculation. Ejaculation (EN), mount (MN), or intromission (IN) cumulative numbers [i.e., ejaculation frequency (EF)] were also estimated, by adding up the number of ejaculations, mounts or intromission recorded during the six copulatory tests. We then phenotyped males based upon EL, as previously described (Olivier et al., 2006). Rapid (EL < 300 s; 20%), normal (EL 300–600 s; 46%), slow (EL > 600–900 s; 22%) or sluggish (EL > 900 s; 12%) copulators were identified and grouped them accordingly; keeping this phenotyping is important because the present work intends to challenge those published earlier (Pattij et al., 2005; Olivier et al., 2006; Olivier and Olivier, 2019). We also assayed EN as a copulatory variate of phenotyping (Pattij et al., 2005; see also Supplementary Material). When EN was used to phenotype, male rats were identified as very high (VH; >20 ejaculations, 12%), high (H; 16–19 ejaculations, 20%), average (AV; 11–15 ejaculations, 40%), low (L; 6–10 ejaculations, 20%) and very low (VL; 1–5 ejaculations, 8%) ejaculators.

### Quantitative Polymerase Chain Reaction (qPCR)

#### Sample Collection

All male rats were anesthetized and sacrificed by decapitation, just a week after their last copulatory encounter took place. The brains were rapidly removed and frozen. The AMG, OB, MPOA, and VMH were all dissected according to previous procedures (Antaramian et al., 2015). Brain samples were each immersed in separate sterile, RNase-free microfuge tubes containing 300 μl of TRIzol (Life Technologies, Carlsbad, CA, United States) and stored at −70°C. RNA extraction was achieved following the manufacturer’s recommendations (Life Technologies). RNA integrity was assessed by electrophoresing samples through 1% agarose gels. RNA concentration was estimated through spectrophotometry (Nano Drop 2000; Thermo Scientific; Wilmington, DE, United States).

#### Single Strand Complementary DNA (cDNA) Synthesis

cDNA synthesis was achieved by using the Moloney Murine Leukemia Virus (M-MLV) Reverse Transcriptase Kit from Promega (Madison, WI, United States). Briefly, we prepared a working solution containing (per reaction): total RNA (2 μg), oligo dT (50 μM; Roche) and random primers (1.2 mM; Roche) diluted in RNase free water (final volume of 15 μl). The reaction tubes were heated at 65°C for 5 min, then cooled on ice and supplemented with the reaction mix (10 μl) containing dNTP mix (10 mM), M-MLV reaction buffer 5X (5 μl), M-MLV (200 U) diluted in RNase free water (final volume 25 μl). All the reaction tubes were incubated at 25°C for 10 min. The temperature used for cDNA synthesis was 55°C for 30 min; cDNA samples obtained were then stored at −20°C.

#### qPCR Protocol

We estimated the expression levels of ESR1, PR, AR, CPY19, DNMT3a, and DNMT1 genes by implementing qPCRs using primers designed based on the sequences reported for each gene in the Probe Finder Data Base (Table 1; Universal Probe Library Roche Mannheim, Germany). Tyrosine 3-monooxygenase/tryptophan 5-monooxygenase activation protein zeta was used as the housekeeping gene (Norm Finder software; Department of Molecular Medicine Aarhus University Hospital, Denmark). Quantitative PCRs were conducted per area in each rat. To make them comparable throughout the population, we always used equal amounts of cDNA across different brain areas and individuals; non-diluted and diluted 1:10, 1:100, 1:1000, 1:10000 standards were used to better calibrate the assays. Also, because we wanted to evaluate the behavior of individual data sets relative to the entire population, the results of each rat were compared versus those obtained from a single standard per area produced by the pooling of all of the corresponding samples (Pfaffl, 2001). All qPCR reactions were performed in duplicate using a 96-well plate format (Light Cycler 480; Roche). Each reaction tube was filled with a solution containing cDNA (2 μl), forward and reverse primers (oligo T4; 300 nM), Light Cycler 480 Probes Master Mix 2X (5 μl; Roche), Probe (1 μM; Roche), diluted in molecular grade water (final volume10 μl). The 2^–ΔΔCt^ method was used to estimate relative levels of gene expression (Pfaffl, 2001). The qPCR conditions used for each gene are shown in Table 1. Lastly, it is worth mentioning that the expression of the genes selected here has been previously considered as sexually relevant since they are assumed to underlie, at least in part, differences in sexual performance observed between copulating and non-copulating male rats (Antaramian et al., 2015). In this work, we instead targeted EL- (rapid, normal, slow, and sluggish) or EN- (VH, H, AV, L, and VL ejaculators) phenotyped copulating males.

**TABLE 1:** PCR probe sequences and amplification conditions.

**Gene (No. GenBank)**		**Oligonucleotides**	**Size product**	**Probe No.^2^**	**Ta°C**	**Dilution**	**Efficiency**	**Slope**
3-Monooxygenase/tryptophan 5-monooxygenase activation protein zeta	Ywhaz BC094305	5′-ctaccgctacttggctgagg-3′ 3′-tgtgactggtccacaattcc-5′	63 nt^1^	9	60 58	Non-diluted	2.3 2.1	−2.7 −3.1
Androgen receptor	AR NM_012502.1	5′-ggcgcttctaccagctca-3′ 3′-gaattgatgcagctctcttgc-5′	68 nt	128	60	1:10	2.2	−2.7
Estrogen receptor alpha	ESR1 NM_012689.1	5′-tttctttaagagaagcattcaagga-3′ 3′-ttatcgatggtgcattggttt-5′	72 nt	130	60	Non-diluted	2.1	−3.0
Progesterone receptor	PR NM_022847.1	5′-ggcagctgctttcagtagtca-3′ 3′-tggtcatcgatgtgtaagttcc-5′	70 nt	53	58	1:10	1.9	−3.5
Aromatase	CYP19 NM_017085.2	5′-ggaaatccacactgttgttgg-3′ 3′-tgaagttttccaccactttcaa-5′	77 nt	9	60	Non-diluted	2.0	−3.1
DNA methyl-transferase 1	DNMT1 NM_053354.3	5′-aactcgtcttggtttgagacct-3′ 3′-gcgactgcaatacacactgaa-5′	75 nt	55	60	1:100	1.8	−3.7
DNA methyl-transferase 3a	DNMT3a NM_001003958.1	5′-aacggaagcgggatgagt-3′ 3′-actgcaatcaccttggcttt-5′	70 nt	75	58	1:100	2.1	−2.9

*^1^Nucleotides. ^2^Probe number for Universal Probe Library Roche.*

### Statistical Analysis

**FIGURE 1:**
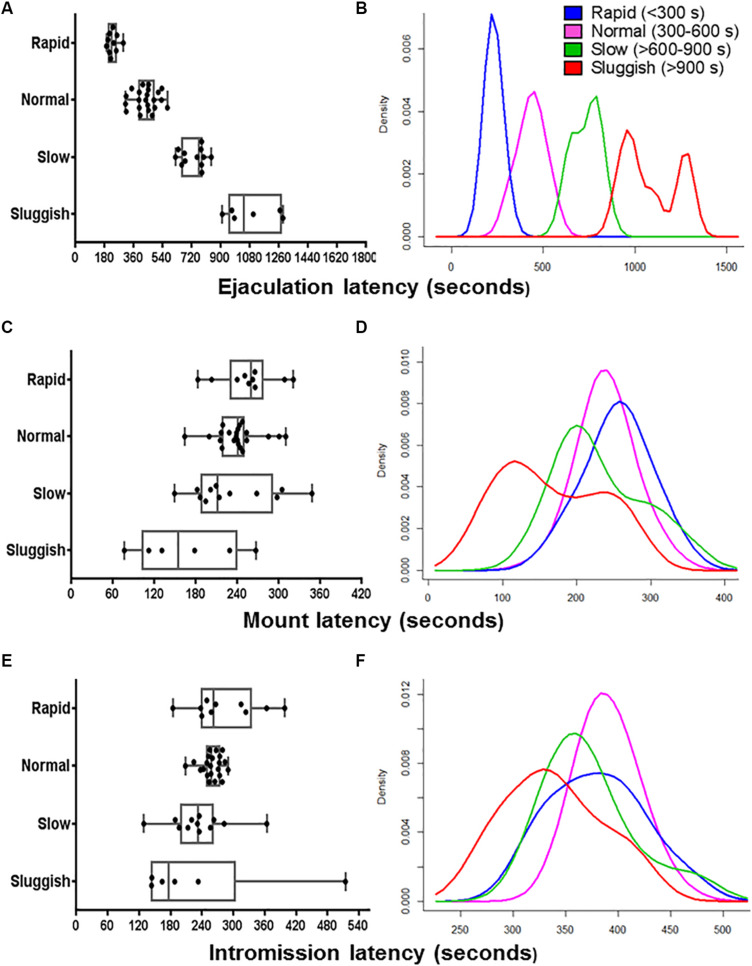
The internal consistency of the presumed EL-ejaculatory endophenotypes is compromised by introducing any other copulatory parameter in addition to EL as phenotyping variate. Boxplots **(A,C,E)** and probability density plots **(B,D,F)** allowed us to evaluate the internal consistency of the presumed EL-ejaculatory endophenotypes **(A,B)** after introducing as covariates of phenotyping mount latency **(C,D)** and intromission latency **(E,F)**. When EL was used as the exclusive phenotyping variate, a clear-cut segregation with virtually no overlapping across the presumed EL-ejaculatory endophenotypes was observed **(A,B)**. In contrast, when EL was paired with mount latency **(C,D)** and intromission latency **(E,F)**, a great deal of overlapping occurred among the presumed EL-ejaculatory endophenotypes.

The results reported were obtained from two cohorts of 25 male rats each. Sexually naïve, non-phenotyped males (*n* = 50) were tested in six copulatory encounters (see copulatory test and male categorization in the Materials and Methods section) aimed at classifying them based on EL or EN, as previously reported (Pattij et al., 2005). We recorded, per male rat, the following copulatory parameters: latencies of mount, intromission and ejaculation (in seconds), and the accumulated number of mounts, intromissions and ejaculations, for each ejaculatory series performed in every copulatory test, each one spanning 30 min. We first evaluated the distribution of copulatory parameters across the population through Shapiro–Wilk’s test (Supplementary Figure S1). As shown in Supplementary Figure S1, ejaculation latency (EL) and intromission latency (IL) did not pass the normality tests (*p* > 0.05). For this reason, no-parametric tests (Kruskal–Wallis and *U*-Mann–Whitney) were used later to evaluate differences between defined endophenotypes when covariates of phenotyping were added (see below). The copulating male rats were classified as rapid (EL < 300 s), normal (EL 300–600 s), slow (EL > 600–900 s) or sluggish (EL > 900 s) based upon the EL mean value obtained after averaging all ejaculatory series per male. Also, each copulating male rat was phenotyped as very high (>20 ejaculations), high (16–19 ejaculations), average (11–15 ejaculations), low (6–10 ejaculations), and very low (1–5 ejaculations) according to the number of cumulative ejaculations across the copulatory tests. The overall distribution around the measure of central tendency and the internal consistency of the EL- or EN-ejaculatory endophenotypes was first tested by introducing as covariates of phenotyping any other of the copulatory parameters (IL, ML, IN, and MN); the relationships between EL- or EN- endophenotypes and each covariate were represented through boxplots and probability density plots (Figure 1A for EL-phenotyped rats and Supplementary Figure S2B for EN-phenotyped rats). We then applied Kruskal–Wallis’ tests to evaluate inter-phenotype overlapping, an indicator of inter-phenotype inconsistency, considering a *p*-value less than 0.001. Then, we used *U*-Mann–Whitney’s tests to evaluate whether a particular endophenotype differed significantly from the preceding or the coming endophenotype within the order sequence (e.g., rapid vs. normal, normal vs. slow, and slow vs. sluggish). Only EL or EN classification has significant differences among pair comparisons of endophenotypes. To test further the internal consistency of the presumed EL- or EN-ejaculatory endophenotypes, we examined the endophenotype stability after introducing the entire set of interactions established among all copulatory parameters considered in the study by using principal component analyses (PCA). Since the copulatory parameters were measured in seconds or frequency, we decided to center and scale the data set. The results were graphed in PCA biplots (Figure 3 for EL-phenotyped rats and Supplementary Figure S3 for EN-phenotyped rats). Additional PCA-backup analyses are presented in Supplementary Figure S5. Lastly, because all the previous analyses indicated that the presumed EL- or EN-copulatory endophenotypes progressively lose internal consistency as the number of copulatory parameters introduced in the analyses increases, we decided to evaluate the degree of similarity (clustering analysis based on Euclidean distance) among the previously EL- or EN- phenotyped copulating males by using a complete-linkage dendrogram. After this, we identified the percentage of presence of the different phenotypes (covariate) in every cluster (Figure 4A for EL-phenotyped males and Supplementary Figure S4A for EN-phenotyped males). We also evaluated the degree of similarity (clustering analysis based on Euclidean distance) among EL-or EN-phenotyped copulating males by combining complete linkage cluster dendrograms and heat map for all the relative expression levels of ESR1, PR, AR, CPY19, DNMT3a and DNMT1 in AMG, MPOA, VMH and OB (Figure 4B for EL-phenotyped males and Supplementary Figure S4B for EN-phenotyped males). Again, before entering the data into the analyses, all units used to record copulatory parameters and relative gene expression levels were centered and scaled. The complete linkage clustering technique employed here uses an agglomerative nesting clustering algorithm in which each object is initially considered as a single-element cluster. At each step of the algorithm, the two clusters that are the most similar are combined into a new bigger cluster to form nodes. This procedure is iterated until all points are member of just one single big cluster known as root. The result is a tree build up from the bottom to the top that can be plotted as a dendrogram. In this way, the analysis computes all pairwise similarities across the elements placed in different clusters along the hierarchy, and considers the maximum value of these similarities as the measured of the distance between clustered pairs of observations. To combine heat maps with complete linkage cluster dendrograms, we executed the script reported in the following link: https://github.com/ItztliSanchez/Trejo-Sanchez-2020.git. We also used Graph Pad Prism 6 software (La Jolla, CA, United States^1^) to generate boxplots and the other statistical analyses were computed and graphed using R Core Team (2020). The entire data sets are publicly available in Trejo-Sánchez, Itztli (2020): Dataset of male rat copulatory behavior. figshare. Dataset. 10.6084/m9.figshare.12108984.v1.

## Results

### Phenotyping Using a Single Copulatory Parameter Masks Internal Inconsistencies of Male Copulatory Endophenotypes

**FIGURE 2:**
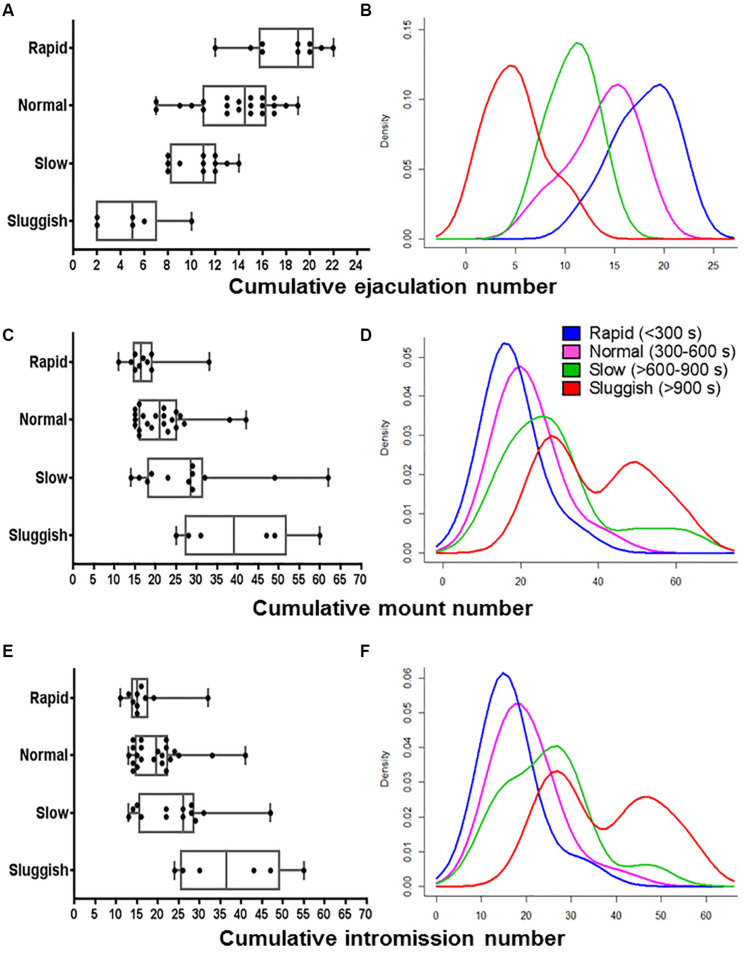
The internal consistency of the presumed EL-ejaculatory endophenotypes is compromised by introducing any other copulatory parameter in addition to EL as phenotyping variate. Boxplots **(A,C,E)** and probability density plots **(B,D,F)** allowed us to evaluate the internal consistency of the presumed cumulative ejaculation number **(A,B)**, cumulative mount number **(C,D)**, and cumulative intromission number **(E,F)**. In contrast, when EL was paired with cumulative mount number **(C,D)** and cumulative intromission number **(E,F)**, a great deal of overlapping occurred among the presumed EL-ejaculatory endophenotypes. Something similar happened when El was paired with cumulative ejaculation number **(A,B)**, albeit the degree of overlapping among the presumed EL-ejaculatory endophenotypes was smaller.

To evaluate the internal consistency of the copulatory endophenotypes, we analyzed the distribution of rapid, normal, slow, and sluggish male rats after introducing ML, IL, EN, MN, or IN as co-variates. As shown in the boxplot and probability density plot showed in Figures 1, 2, EL-endophenotypes were not internally consistent since virtually all of them become overlapped when any of the other copulatory parameters were introduced in the analyses. With regard to the hypothesis that ejaculatory endophenotypes exist, even though Kruskal–Wallis’ tests showed statistically significant differences (*p* **<** 0.001) among all EL- or EN-copulatory endophenotypes, *U*-Mann–Whitney’s tests showed no statistically significant differences when a specific endophenotype is compared with the preceding or the coming endophenotypes along the ordered sequence. Accordingly, sluggish and slow, slow and normal, normal and rapid were not significantly different (*p* **>** 0.001) for the four copulatory parameters considered (IL, ML, IN, and MN) in the analyses. A similar result was observed in EN-phenotyped males (Supplementary Figure S2). Thus, phenotyping based on a single, optimized copulatory parameter of reference over-reduces the complexity of male’s copulatory behavior, thus leading to uncertain conclusions.

**FIGURE 3:**
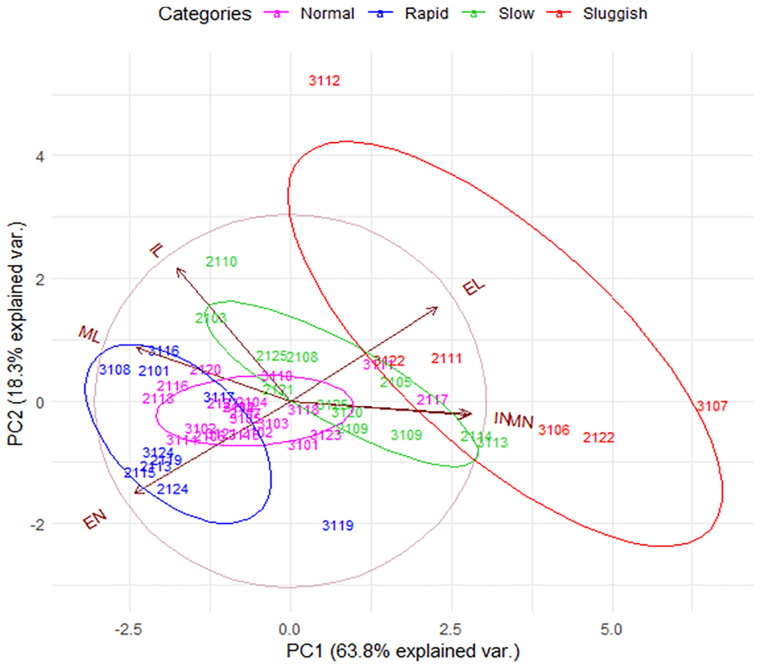
The internal consistency of the presumed EL-ejaculatory endophenotypes is compromised by introducing the entire set of copulatory parameters to phenotype copulating males. Principal component analyses biplot that allowed us to evaluate the internal consistency of the presumed EL-ejaculatory endophenotypes after introducing all copulatory parameters as covariates of phenotyping. Notice that the way copulatory parameters interact one another per copulating male rat tends to be similar regardless of the EL-ejaculatory phenotype each were assigned to. This circumstance leads to a distribution characterized by a strong overlapping of copulating male rats assigned to either of the presumed EL-ejaculatory endophenotypes; most of them share the same space in the graph (gray circle). The exception being a handful of sluggish copulating males. However, the phenotypic variation among them is so high, that envisioning them as representing true endophenotypes is untenable; they might be better seen as outliers. Also notice that 82.1% of the population variance was explained by PC1 and PC2. Copulating male rats assigned to the presumed EL-ejaculatory endophenotypes are numbered and color-coded differentially; the color-key is placed at the upper edge of the PCA biplot. Copulating male rats assigned to the same ejaculatory endophenotype are enclosed by elliptical traces of the same color; the greater the ellipse area, the highest the estimated intra-categorical variability. Vectors represent copulatory parameters; the closer the angle between vectors, the higher their correlation. Complementary information on PCA is showed in Supplementary Figure S5. PC1: Principal component one; PC2: Principal component two. EL, ejaculation latency; EN, cumulative ejaculation number; IL, intromission latency, IN, cumulative intromission number; ML, mount latency; MN, cumulative mount number.

To further evaluate the internal consistency of EL-based male phenotyping, we conducted a PCA in which all copulatory parameters were introduced as variables. In PCA plots, the relative position of each EL-endophenotyped male throughout the graphical space and his relatedness to the rest of the other males across the sampled population is defined by the interactions of the copulatory variables established among them. As seen in Figure 3, even though EL-phenotyped males tend to form loose groups across the graphical space, 92% of them share a common probabilistic space. The exceptions are four out of six sluggish males that comprise the 8% of the sample. A similar result was observed after phenotyping males based on their EN (Supplementary Figure S3). Hence, although there might be copulator males whose phenotypes might indeed group closely when classified based on the EL or EN, male rat copulatory behavior is much more variable with males showing various degrees of “short to delayed” ELs and/or of “reduced to high” ENs. Possibly, this diversity will even be greater as more parameters (e.g., individual gene expression patterns or individual sex hormones levels) are added to the analyses. As our results stand, it seems that male copulatory endophenotypes are not separate entities. Instead, the variability of copulatory behavior across copulator males suggests that their individual phenotype arise from a common phenotypic plan.

### Relative Expression of Sexually Relevant Genes in Brain Limbic Areas Is Not Endophenotype Specific

As in previous studies (Pattij et al., 2005), in our own, male rats were first phenotyped and grouped using EL as the copulatory variable of phenotyping, after conducting six copulatory encounters. Endophenotypes thus defined had nearly no overlap (Figure 1A). When male rats were phenotyped using EN as the copulatory variable of phenotyping, however, inter-endophenotype overlapping was much greater (Supplementary Figure S4A). Then, EL- or EN- endophenotypes seem more alike than different. So we evaluated the degree of similarity among phenotyped male rats including all the copulatory parameters through complete-linkage dendrograms. We found, again, that males presumably having different endophenotypes sparse across the clusters. We also contrasted the percentage of males from the same endophenotype that consistently clustered together. We clustered both EL- or EN-phenotyped male rats up to the Euclidean distance of 4 in height. This cut point left out of the analyses the two atypical values, while keeping the number of groups at endophenotypes comparable. In both cases, there were a total of six clusters. For EL- phenotyping only the fifth cluster (from left to right), represented 90% (9 of 10) of rapid males. However, the same cluster shared 64% (14 of 22) of normal males. For the case of EN-phenotyped males, the fifth cluster comprised 100% (6 of 6) of the VH male rats, 90% (9 of 10) of H males and 40% (8 of 20) of AV male rats. These results corroborate the inconsistency of the presumed EL- or EN- endophenotypes and of the covariates of phenotyping.

**FIGURE 4:**
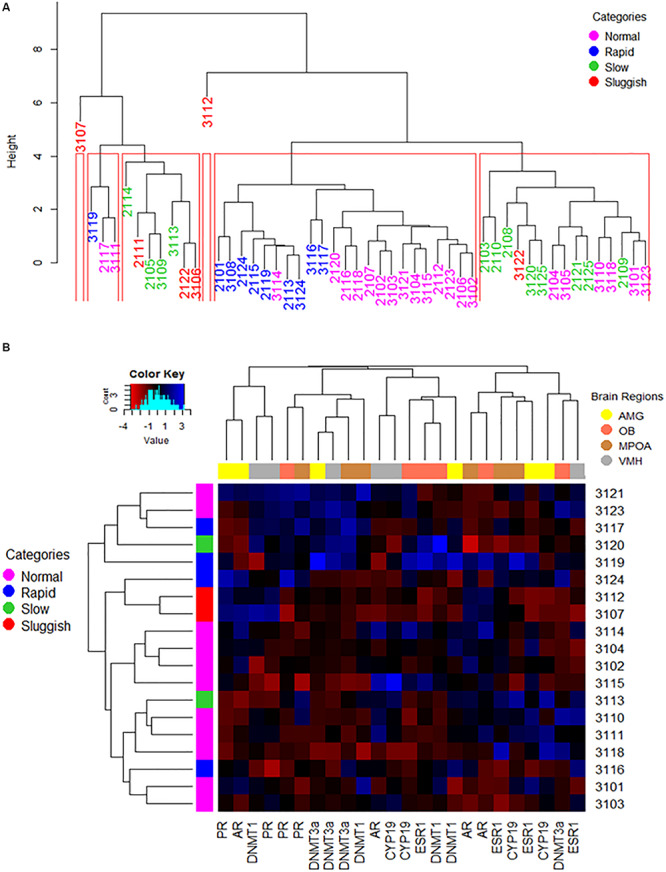
The way copulatory parameters and the expression of copulation relevant genes in pertinent brain limbic areas interact one another or altogether are not specific to either of the presumed EL-ejaculatory endophenotypes. **(A)** A complete linkage clustering dendrogram was used to estimate the degree of similarity among EL-phenotyped copulating males based on the way copulatory parameters interacted one another per phenotyped rat across the entire population of EL-phenotyped males (*n* = 50). Notice that the population, including some sluggish copulating male rats, threshes mixed down through the branching pattern of the dendrogram until reaching the tip of the tree where individual rapid, normal slow and sluggish copulating males show different degrees of phenotype similarity depending upon the final location within the tree, and likely the frequency of each “endophenotype” across the population. In red, we show the animal clusters obtained at the cut point of 4 in height. Such clusters were used to compare the consistency of the EL-ejaculatory endophenotypes. Overall, the way copulatory parameters interact one another in most of the copulating males are more alike than distinct regardless of EL-ejaculatory endophenotype each were assigned to. **(B)** The combined used of a heat map and of an agglomerative complete linkage clustering dendrogram allowed us to evaluate the degree of similarity among EL-phenotyped copulating males based on the way brain regional patterns and levels of gene expression interacted with one another per phenotyped rat across the entire population of EL-phenotyped males (*n* = 20). In this figure, the heat map color-codes (blue the highest levels; see color key on the upper left corner) the relative levels of expression of AR, ESR1, CYP19, PR, DNMT1 and DNMT3a genes, clustered based upon the degree of similarity per limbic structure (see upper right corner for the color code assigned to each region in the dendrogram placed at the upper border of the heatmap) and per EL-phenotyped male (dendrogram placed at the left side of the heat map). Numerals on the right correspond the numeric code assigned to each of the EL-phenotyped male, also color-coded based upon the presumed endophenotype assigned (see upper right corner in **A**). Notice that gene expression clustering across the EL-phenotyped male population is ejaculatory endophenotype independent. The histogram on the upper left corner depicts the overall distribution of gene expression levels across the population of EL-phenotyped males. Amygdala: AMG; Olfactory bulb: OB; Medial preoptic area: MPOA; Ventromedial hypothalamus: VMH.

As the conventional perspective assumes that EL-endophenotypes arise from brains displaying phenotype-specific attributes (Olivier et al., 2006; Olivier and Olivier, 2019), we next tested whether the expression of ESR1, PR, AR, CPY19, DNMT3a, and DNMT1 genes in the AMG, OB, MPOA, and VMH formed endophenotype specific clusters. In contrast to predictions, no endophenotype specific gene assemblages were observed as analyzed by complete-linkage, clustering dendrograms (Figure 4A). A similar result was observed after phenotyping males based on their EN (Supplementary Figure S4A).

We built also a clustering dendrogram aimed at evaluating whether EL- or EN-phenotyped males displayed endophenotype-specific assemblages of gene expression across limbic areas. Overall, we found four large gene clusters (Figure 4B and Supplementary Figure S4B). The first cluster grouped AMG and VMH having similar relative gene expression levels of AR-PR and PR-DNMT1, respectively, across the copulating male’s population. The second cluster showed that all limbic areas analyzed had similar PR and DNMTs relative gene expression levels, across the copulating male’s population. The third cluster showed that relative gene expression levels of AR-CYP19 in VMH, ESR1-CYP19 and DNMT1 in OB and AMG were similar across copulating male’s population. Lastly, the fourth cluster grouped OB/MPOA and AMG/MPOA having similar relative gene expression levels of AR and ESR1-CYP19, respectively, regardless of the copulatory endophenotype. Overall, no copulatory endophenotype specific gene expression clustering was observed across limbic structures.

## Discussion

In man and in some other mammals, female and male hetero-, homo-, bi-, a-, hyper-, hypo-, trans- and other expressions of sexuality are thought to represent distinct phenotypes defined not only by their sexual behavioral display and sexual preference, but by brains having phenotype specific functional morphological attributes and genetic/epigenetic architectures (see introductory remarks). In following this tradition, it has been suggested that, at least in heterosexual copulating men and male rats, EL or ejaculation frequency (i.e., in this work ejaculation number; EN) represent each, endophenotypic traits that lie on the causal pathway between the genes, the brain and the disorders of ejaculation (Pattij et al., 2005; Waldinger and Olivier, 2005; Olivier et al., 2006; Ventura-Aquino and Paredes, 2017). In addition, it is believed that ejaculatory endophenotypes are genetically imbedded and hence heritable and state independent (e.g., Pattij et al., 2005; see also introductory remarks). These claims, however, are challenged by past and recent evidence that supports that sexual behavior is highly variable and that the brain sexual functional morphology represents an intricate sexual phenotypic mosaic (see introductory remarks). Also, we have previously argued that the presumed ejaculatory endophenotypes are not so, but that they represent adaptive traits developed to afront ecological challenges (Lucio et al., 2012) and, as such, they might be mutable under precise state pressures (e.g., Rodríguez-Manzo and Canseco-Alba, 2014; Rodríguez-Peña et al., 2017; Canseco-Alba and Rodríguez-Manzo, 2019), regardless of whether these pressures are intrinsic or extrinsic to the organisms. Hence, for the ejaculatory endophenotypes to be considered a valid biological ground to understand normal or abnormal ejaculation within male populations, they must pass a validation test by assessing their internal consistency. A strategy to do this is to examine first whether the presumed endophenotypes retain or lose their identity when subjected to state pressures; by definition endophenotypes must be state independent (Flint and Munafò, 2007; Walters and Owen, 2007). Here, we approached the state-dependence property of the presumed ejaculatory endophenotypes by assessing first the effect of introducing as covariates of phenotyping ML, IL, EN, MN, or IN for EL-phenotyped copulating males or EL, ML, IL, MN, or IN for EN-phenotyped males, on endophenotype stability. Our results showed that including any other copulatory parameter, in addition to EL or EN, as phenotyping covariate, compromises the stability of the presumed EL- or EN-ejaculatory endophenotypes. A single attribute, or pairs of them, might not define fully an endophenotype though. Instead, it might be that summing up the effects of various traits could strengthen each endophenotype’s consistency (Flint and Munafò, 2007; Walters and Owen, 2007). We then tested this possibility by evaluating the effects of introducing all of the copulatory parameters recorded as variables of phenotyping on the stability of the presumed EL- or EN-ejaculatory endophenotypes. The stability of the presumed EL- and EN- ejaculatory endophenotypes also failed at withstanding the test of state independence. Hence, at least at the behavioral level, our results support that the presumed EL- or EN-ejaculatory phenotypes lack intrinsic consistency since increasing the number of variables introduced in the analyses, greatly erodes their stability across the population of copulating males, the latter represented by the extensive overlapping of the copulating male rats assigned to either of the presumed EL- or EN-ejaculatory endophenotypes. In other words, the copulating males studied here display a mosaic-like copulatory behavioral pattern that may include attributes, in different degrees for each copulating male, assigned to any of presumed EL- or EN-ejaculatory endophenotypes. So, even though the concept sustaining the existence of male ejaculatory endophenotypes might useful in some clinical settings (see Walters and Owen, 2007 for considerations on this topic with regard to the use of the endophenotype concept in psychiatry), as things stand now, its use might be misleading if sustained based only upon a single copulatory or ejaculatory parameter having no endophenotype specific gene architectures identified yet.

A fundamental assumption with regard to endophenotypes, is that their variation depends upon the additive effects of a reduced number of genes each contributing with a small effect to the endophenotype. Theoretically, the presumed genetic simplicity of endophenotypes would make them more genetically tractable in comparison to the complexity of the entire phenotype (see Walters and Owen, 2007 for a discussion on this topic), whether diseased or not. Another basic premise with regard to endophenotypes, at least from a psychiatric stand, is that they should have reliable and valid psychometric and neurometric properties and be sufficiently sensitive to pinpoint individual differences (also see Walters and Owen, 2007 for thoughtful considerations on the matter). Based on these premises, because copulatory behavior arises from the conjoint work of various brain centers, we thought important to evaluate the internal consistency of the presumed EL- and EN-ejaculatory endophenotypes by looking at the patterns and levels of expression of a handful of copulation relevant genes in a few copulation relevant brain limbic areas. Purposely, we assessed whether these parameters were specific to any of the presumed EL- or EN-ejaculatory endophenotypes. In contrast to predictions, we found ESR1, PR, AR, CPY19, DNMT3a, and DNMT1 gene expression across the AMG, OB, MPOA, and VMH to be uncorrelated with any of the presumed EL- or EN-ejaculatory endophenotypes. Gene expression patterns and levels overlapped across the population of EL- or EN- phenotyped copulating males. Hence, together, the behavioral and molecular data gathered here show that presumed EL- or EN-ejaculatory endophenotypes have no intrinsic consistency. Instead, it appears that animals presumed to display distinct ejaculatory endophenotypes in fact represent variations of a common copulatory phenotype. In support to this conclusion, notice that most of the EL- or EN-phenotyped copulating males cluster within the same area of the PCA-biplots. Hence, even though we found that there were group-level differences in all the variables that were studied, these differences did not add-up to create the presumed, EL- or EN-ejaculatory endophenotypes.

Attributing significant variations of phenotypic traits to gene mutations has become a customary, problem-solving strategy in biological/biomedical sciences. The endophenotype’s notion is at the core of this trend (Gottesman and Gould, 2003; Burmeister et al., 2008; Garcia et al., 2010; Iacono et al., 2014; Zietsch et al., 2015). Sizeable phenotypic variations, nonetheless, may also emerge through epigenetic mechanisms (Ayala-García et al., 2013; see also comments on the effects of epigenetics on endophenotype expression in Hasler et al., 2006). That this might be the case for the copulator male rats included in our sample is supported by data showing that relative gene expression levels of DNMT-1 and DNMT-3a differ greatly across individuals; these enzymes are involved in the transferring of methyl groups to DNA and thus in the epigenetic regulation of gene expression. Also, the lack of internal consistency of the presumed EL- or EN-ejaculatory endophenotypes, the degree of interindividual variation observed among copulating male rats assigned to any of the presumed EL- or EN-ejaculatory endophenotypes, and the high degree of similarity across the EL- or EN-phenotyped copulating male rats, also pinpoint to epigenetic processes as the likely source of variation. Nugent et al. (2015, 2017) have in fact demonstrated that adult male sexual behavior in male mice and rats requires several genes to be de-methylated in neurons of the developing preoptic area (see also Mosley et al., 2017). Although the epigenetic factor involved in generating these inter-individual variations is unclear, a good candidate worth exploring is the mother-litter, differential care. Indeed, it has been shown that genital licking by the mother early during postnatal development influences copulatory behavior in adulthood; the lengthier the liking, the better the display of adult copulatory behavior (Lenz and Sengelaub, 2006, 2009, 2010). The merits of this idea must be addressed in future experiments.

To end the discussion section of this work, we would like to make three additional considerations and a final cautionary note. First, why would it be best for any species interests to have a no endophenotype-mediated relationship between brain and behavior with regard to sex? A recent study suggests that keeping phenotype, sexually dimorphic features may have negative effects for the species survival (Martins et al., 2018). Second, it has been suggested that males have the ability to adjust the quality of the ejaculation based upon the risk of or the actual sexual competition (Parker and Pizzari, 2010). Models predict that males adjust following the same rules based on the presumption that they have similar copulatory abilities and therefore strategies to solve the conflict (Parker, 1998). The fact that copulatory behavior seems not to be endophenotypic, but highly diverse, suggests that assumptions of these models may be incorrect. Third, neuro-pharmacologists dealing with “sexual dysfunctions” used the concept of ejaculatory endophenotypes (Olivier et al., 2006) to dictate guidelines to develop pharmacological agents to treat these conditions. Since the presumed ejaculatory endophenotypes seem to lack intrinsic consistency, their use as a conceptual frame to design pharmacological agents must be taken with reserve. Finally, it is worth recognizing that the present work has limitations that restrict, to some degree, the breath of its conclusions. Future studies must then explore (1) the heritability of the presumed EL- or EN-ejaculatory endophenotypes, (2) other behavioral, genetic and neurophysiological traits with the potential of being endophenotypic characters, (3) expression of other copulation/ejaculation relevant genes in a greater number of brain areas, and (4) the effects of increasing further sample size to more thoroughly evaluate the existence of male ejaculatory endophenotypes. In spite of our work limitations, nonetheless, the fact that sexual training (i.e., increasing the extrinsic state pressures) lengthens ejaculation latency in copulating male rats displaying short ejaculation latencies (Rodríguez-Peña et al., 2017) supports that ejaculatory latency is not an endophenotypic trait since it is not state independent. Finally, even if ejaculation latency would satisfy the criteria to be considered an endophenotypic trait, this would not exclude the possibility that it is an epiphenomenon with respect to the condition such endophenotype trait is presumed to represent (Walters and Owen, 2007).

Overall, our results provide cautionary information on the utility of the notion of endophenotypes to study male copulatory behavior in copulating heterosexual subjects. The diversity of male copulatory behavior observed in male rats here is in line with current ideas suggesting that sexuality is better perceived as a non-linear, behavioral manifestation that arises from combinatorial, muti-morphic mosaics of brain molecular architectures and functional morphological arrangements. Under this context, direct predictions about how sexual behavior will be manifested based on brain organizational features are condemned to serious flaws.

## Data Availability Statement

The datasets generated for this study can be found in the Universal Probe Library Roche. Dataset of male rat copulatory behavior. doi: 10.6084/m9.figshare.12108984.v2. https://github.com/ItztliSanchez/Trejo-Sanchez-2020.

## Ethics Statement

The animal study was reviewed and approved by the Comisión Institucional para el Cuidado y Uso de Animales del Laboratorio, IIB, UNAM (Permit No. 163).

## Author Contributions

IT-S and GG-O designed the research and wrote the manuscript. IT-S and CP-M performed the experiments and analyzed the data. SH-P supervised the statistical analyses and helped for writing the statistical analyses section. All authors contributed to the manuscript revision, read and approved the submitted version.

## Conflict of Interest

The authors declare that the research was conducted in the absence of any commercial or financial relationships that could be construed as a potential conflict of interest.

## Footnotes

1 www.graphpad.com

## Supplementary Material

FIGURE S1 Distribution of copulating male rats before phenotyping, relative to different copulatory parameters. Histogram plots constructed based upon the probability density that depict the distribution of copulator males (*n* = 50), when classified based upon **(A)** ejaculation latency, **(B)** cumulative ejaculation number, **(C)** mount latency, **(D)** cumulative mount number, **(E)** intromission latency, and **(F)** cumulative intromission number. Even though ejaculation and intromission latency curves are slightly right-hand skewed, for the most part, our copulating male sample distributes normally relative to each of the copulatory parameters. So, we can safely say that we begin our study with an unimodal population of copulating male rats.

FIGURE S2 The internal consistency of the presumed EN-ejaculatory endophenotypes is compromised by introducing any other copulatory parameter in addition to EN as phenotyping variate. Boxplots and probability density plots allowed us to evaluate the internal consistency of the presumed EN-ejaculatory endophenotypes **(B)** after introducing as covariates of phenotyping ejaculation latency **(A)**, mount latency **(C)**, intromission latency **(E)**, cumulative mount number **(D)**, and cumulative intromission number **(F)**. When EN was used as the exclusive phenotyping variate, a fair segregation with virtually relatively little overlapping among the presumed EN-ejaculatory endophenotypes was observed **(B)**. In contrast, when EN was paired with mount latency **(C)**, intromission latency **(E)**, cumulative mount number **(D)**, and cumulative intromission number **(F)**, a great deal of overlapping occurred among the presumed EN-ejaculatory endophenotypes. Something similar happened when EN was paired with ejaculation latency **(A)** with the exception of the sluggish copulating males who stayed a bit segregated from the rest of the presumed EN-ejaculatory endophenotypes.

FIGURE S3 The internal consistency of the presumed EN-ejaculatory endophenotypes is compromised by introducing the entire set of copulatory parameters to phenotype copulating males. Principal component analyses (PCA) biplot that allowed us to evaluate the internal consistency of the presumed EN-ejaculatory endophenotypes after introducing all copulatory parameters as covariates of phenotyping. Notice that the way copulatory parameters interact one another per copulating male rat tends to be similar regardless of the EN-ejaculatory phenotype each were assigned to. This circumstance leads to a distribution characterized by a strong overlapping of copulating male rats assigned to either of the presumed EN-ejaculatory endophenotypes; most of them share the same space in the graph (grey circle). The exception being a handful of sluggish copulating males. However, the phenotypic variation among them is so high, that envisioning them as representing true endophenotypes is untenable; they might be better seen as outliers. Also notice that 82.1% of the population variance was explained by PC1 and PC2. Copulating male rats assigned to the EN-ejaculatory phenotypes are numbered and color-coded differentially; the color-key is placed at the right side of the PCA biplot. Copulating male rats assigned to the same ejaculatory endophenotype are enclosed by elliptical traces of the same color; the greater the ellipse area, the highest the estimated intra-categorical variability. Vectors represent copulatory parameters; the closer the angle between vectors, the higher their correlation. Complementary information on PCA is showed in Supplementary Figure S5. PC1: Principal component one; PC2: Principal component two. EL, ejaculation latency; EN, cumulative ejaculation number; IL, intromission latency, IN, cumulative intromission number; ML, mount latency; MN, cumulative mount number.

FIGURE S4 The way copulatory parameters and the expression of copulation relevant genes in pertinent brain limbic areas interacts one another or altogether are not specific to either of the presumed EN-ejaculatory endophenotypes. **(A)** A complete linkage clustering dendrogram was used to estimate the degree of similarity among EN-phenotyped copulating males based on the way copulatory parameters interacted one another per phenotyped rat across the entire population of EN-phenotyped males (*n* = 50). Notice that the population including some very low copulating male rats, threshes mixed down through the branching pattern of the dendrogram until reaching the tip of the tree where individual VH, H, AV, L and VL copulating males show different degrees of phenotype similarity depending upon the final location within the tree, and likely the frequency of each endophenotype across the population. In red, we show the animal clusters obtained at the cut point of 4 in height. Such clusters were used to compare the consistency of the EN-ejaculatory endophenotypes. Overall, the way copulatory parameters interact one another in most of the copulating males are more alike than distinct regardless of EN-ejaculatory endophenotype each was assigned to. **(B)** The combined used of a heat map and of a complete linkage dendrogram allowed us to evaluate the degree of similarity among EN-phenotyped copulating males, based on the way brain regional patterns and levels of gene expression interacted with one another per phenotyped rat across the entire population of EN-phenotyped males (*n* = 20). In this figure, the heat map color-codes (blue the highest levels; see color key on the upper left corner) the relative levels of expression of AR, ESR1, CYP19, PR, DNMT1, and DNMT3a genes, clustered based upon the degree of similarity per limbic structure (see upper right corner for the color code assigned to each region in the dendrogram placed at the upper border of the heatmap) and per EN-phenotyped male (dendrogram placed at the left side of the heat map). Numerals on the right correspond to the numeric code assigned to each of the EN-phenotyped male, also color-coded based upon the endophenotype assigned [see upper right corner in panel **(A)**]. Notice that gene expression clustering across the EN-phenotyped male population is ejaculatory endophenotype independent. The histogram on the upper left corner depicts the overall distribution of gene expression levels across the population of EN-phenotyped males. Amygdala: AMG; Olfactory bulb: OB; Medial preoptic area: MPOA; Ventromedial hypothalamus: VMH.

FIGURE S5 Principal component analyses criteria of design. In our study, we assessed the internal consistency of the presumed ejaculatory endophenotypes by evaluating the distribution of copulating male rats after introducing the way all copulatory parameters interact one another per phenotyped animal, through a principal component analysis (PCA). After evaluating the percentage of variance retained by each principal component **(A)**, we decided to run a two-dimensional analysis because components 1 and 2 explained up to 82.1% of the sample’s variance. This decision was proved to be correct since the values of the square cosine (Cos^2^) for each of the copulatory variables considered in the analysis were all above 0.5 **(B)**, so they all are adequately represented in the sample examined through the two dimensional analysis, Finally, the percentage of contribution of every variable per component was estimated **(C,D)**. The red dashed line in C and D indicates the expected average contribution (16.7%). Having this reference in mind, it becomes clear that mount (ML) and intromission (IL) latencies together with mount (MN) and intromission (MN) cumulative numbers explain most of the sample variance associated to component number one **(C)** and that ejaculation number (EN) and latency (EL) together with mount (MN) and intromission (IN) numbers mainly explain the variance associated to component number two **(D)**. EL, ejaculation latency; EN, cumulative ejaculation number; IL, intromission latency, IN, cumulative intromission number; ML, mount latency; MN, cumulative mount number.
