# Supplementary figures and images for: Male Ejaculatory Endophenotypes: Revealing Internal Inconsistencies of the Concept in Heterosexual Copulating Rats
Source: Front Behav Neurosci. 2020 Jun 26;14:90. doi: 10.3389/fnbeh.2020.00090 (PMC7332778; doi:10.3389/fnbeh.2020.00090)

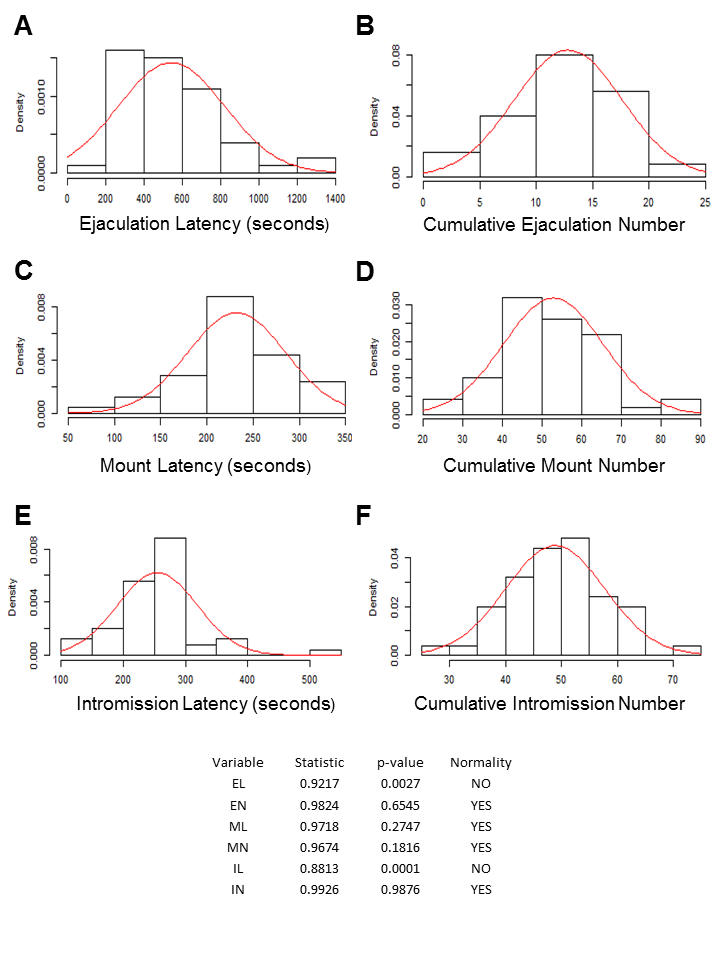

Supplement: FIGURE S1 — Distribution of copulating male rats before phenotyping, relative to different copulatory parameters. Histogram plots constructed based upon the probability density that depict the distribution of copulator males (n = 50), when classified based upon (A) ejaculation latency, (B) cumulative ejaculation number, (C) mount latency, (D) cumulative mount number, (E) intromission latency, and (F) cumulative intromission number. Even though ejaculation and intromission latency curves are slightly right-hand skewed, for the most part, our copulating male sample distributes normally relative to each of the copulatory parameters. So, we can safely say that we begin our study with an unimodal population of copulating male rats. [file Image_1.tif]

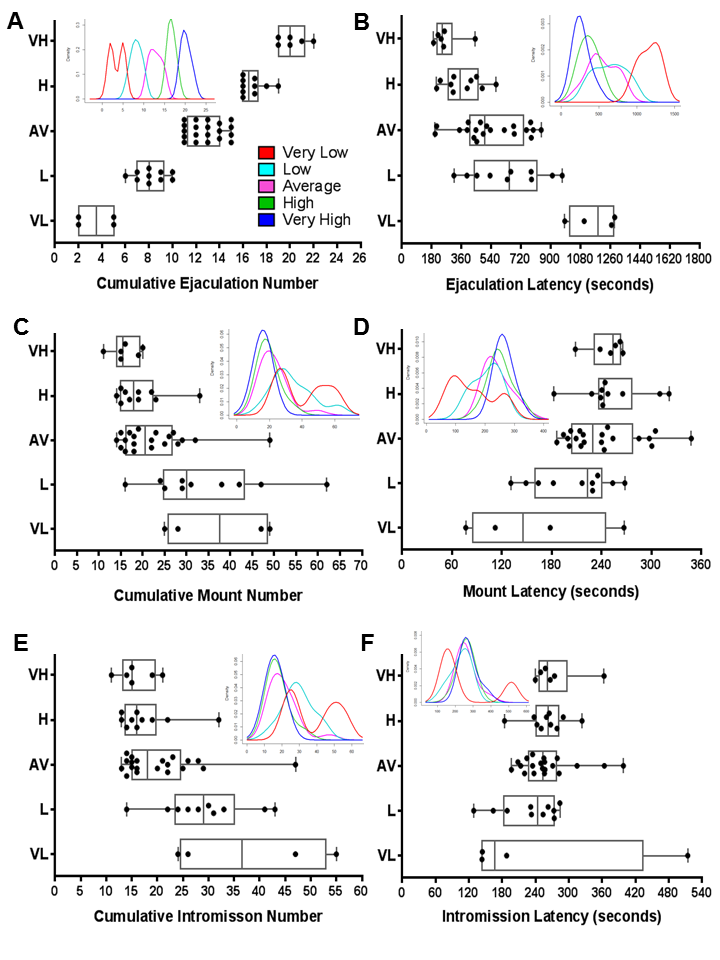

Supplement: FIGURE S2 — The internal consistency of the presumed EN-ejaculatory endophenotypes is compromised by introducing any other copulatory parameter in addition to EN as phenotyping variate. Boxplots and probability density plots allowed us to evaluate the internal consistency of the presumed EN-ejaculatory endophenotypes (B) after introducing as covariates of phenotyping ejaculation latency (A), mount latency (C), intromission latency (E), cumulative mount number (D), and cumulative intromission number (F). When EN was used as the exclusive phenotyping variate, a fair segregation with virtually relatively little overlapping among the presumed EN-ejaculatory endophenotypes was observed (B). In contrast, when EN was paired with mount latency (C), intromission latency (E), cumulative mount number (D), and cumulative intromission number (F), a great deal of overlapping occurred among the presumed EN-ejaculatory endophenotypes. Something similar happened when EN was paired with ejaculation latency (A) with the exception of the sluggish copulating males who stayed a bit segregated from the rest of the presumed EN-ejaculatory endophenotypes. [file Image_2.tif]

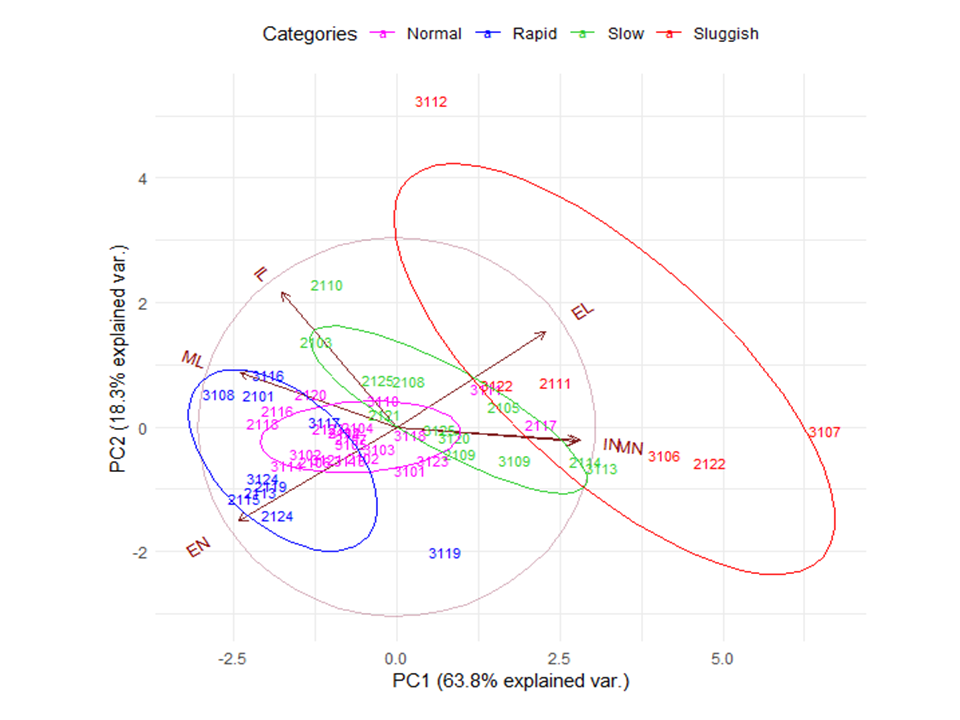

Supplement: FIGURE S3 — The internal consistency of the presumed EN-ejaculatory endophenotypes is compromised by introducing the entire set of copulatory parameters to phenotype copulating males. Principal component analyses (PCA) biplot that allowed us to evaluate the internal consistency of the presumed EN-ejaculatory endophenotypes after introducing all copulatory parameters as covariates of phenotyping. Notice that the way copulatory parameters interact one another per copulating male rat tends to be similar regardless of the EN-ejaculatory phenotype each were assigned to. This circumstance leads to a distribution characterized by a strong overlapping of copulating male rats assigned to either of the presumed EN-ejaculatory endophenotypes; most of them share the same space in the graph (grey circle). The exception being a handful of sluggish copulating males. However, the phenotypic variation among them is so high, that envisioning them as representing true endophenotypes is untenable; they might be better seen as outliers. Also notice that 82.1% of the population variance was explained by PC1 and PC2. Copulating male rats assigned to the EN-ejaculatory phenotypes are numbered and color-coded differentially; the color-key is placed at the right side of the PCA biplot. Copulating male rats assigned to the same ejaculatory endophenotype are enclosed by elliptical traces of the same color; the greater the ellipse area, the highest the estimated intra-categorical variability. Vectors represent copulatory parameters; the closer the angle between vectors, the higher their correlation. Complementary information on PCA is showed in Supplementary Figure S5. PC1: Principal component one; PC2: Principal component two. EL, ejaculation latency; EN, cumulative ejaculation number; IL, intromission latency, IN, cumulative intromission number; ML, mount latency; MN, cumulative mount number. [file Image_3.TIF]

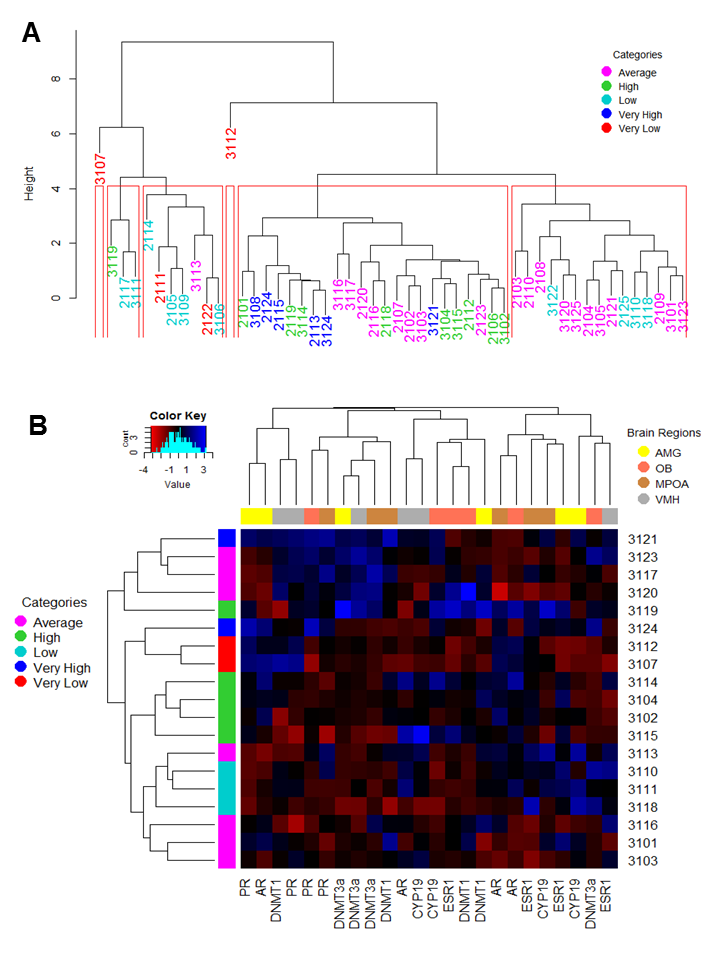

Supplement: FIGURE S4 — The way copulatory parameters and the expression of copulation relevant genes in pertinent brain limbic areas interacts one another or altogether are not specific to either of the presumed EN-ejaculatory endophenotypes. (A) A complete linkage clustering dendrogram was used to estimate the degree of similarity among EN-phenotyped copulating males based on the way copulatory parameters interacted one another per phenotyped rat across the entire population of EN-phenotyped males (n = 50). Notice that the population including some very low copulating male rats, threshes mixed down through the branching pattern of the dendrogram until reaching the tip of the tree where individual VH, H, AV, L and VL copulating males show different degrees of phenotype similarity depending upon the final location within the tree, and likely the frequency of each endophenotype across the population. In red, we show the animal clusters obtained at the cut point of 4 in height. Such clusters were used to compare the consistency of the EN-ejaculatory endophenotypes. Overall, the way copulatory parameters interact one another in most of the copulating males are more alike than distinct regardless of EN-ejaculatory endophenotype each was assigned to. (B) The combined used of a heat map and of a complete linkage dendrogram allowed us to evaluate the degree of similarity among EN-phenotyped copulating males, based on the way brain regional patterns and levels of gene expression interacted with one another per phenotyped rat across the entire population of EN-phenotyped males (n = 20). In this figure, the heat map color-codes (blue the highest levels; see color key on the upper left corner) the relative levels of expression of AR, ESR1, CYP19, PR, DNMT1, and DNMT3a genes, clustered based upon the degree of similarity per limbic structure (see upper right corner for the color code assigned to each region in the dendrogram placed at the upper border of the heatmap) and per EN-phenotyped male (dendro [file Image_4.TIF]

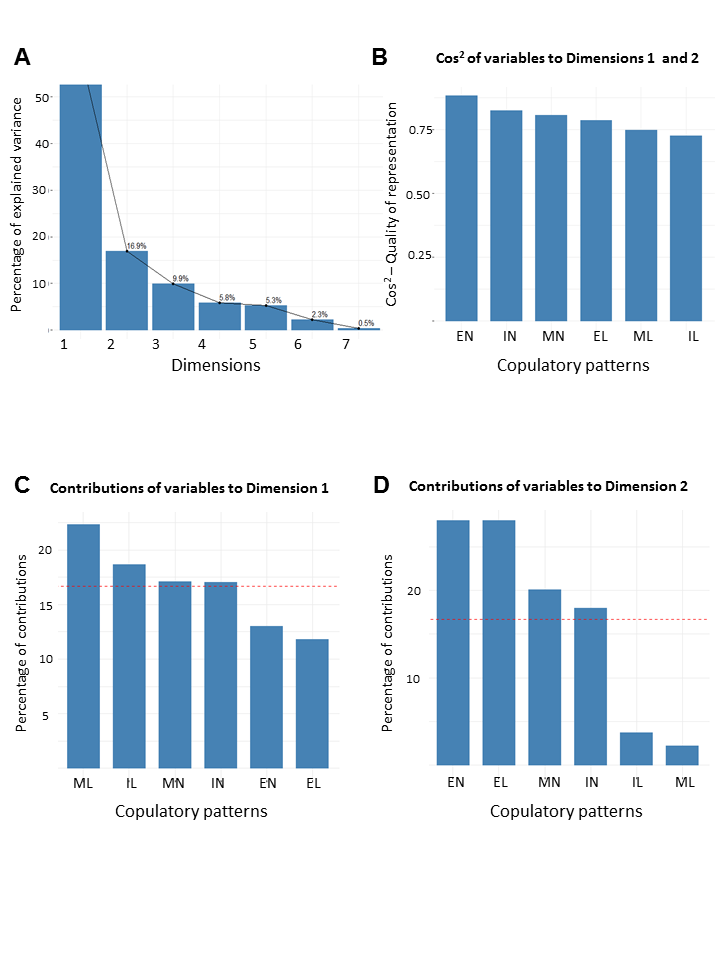

Supplement: FIGURE S5 — Principal component analyses criteria of design. In our study, we assessed the internal consistency of the presumed ejaculatory endophenotypes by evaluating the distribution of copulating male rats after introducing the way all copulatory parameters interact one another per phenotyped animal, through a principal component analysis (PCA). After evaluating the percentage of variance retained by each principal component (A), we decided to run a two-dimensional analysis because components 1 and 2 explained up to 82.1% of the sample’s variance. This decision was proved to be correct since the values of the square cosine (Cos2) for each of the copulatory variables considered in the analysis were all above 0.5 (B), so they all are adequately represented in the sample examined through the two dimensional analysis, Finally, the percentage of contribution of every variable per component was estimated (C,D). The red dashed line in C and D indicates the expected average contribution (16.7%). Having this reference in mind, it becomes clear that mount (ML) and intromission (IL) latencies together with mount (MN) and intromission (MN) cumulative numbers explain most of the sample variance associated to component number one (C) and that ejaculation number (EN) and latency (EL) together with mount (MN) and intromission (IN) numbers mainly explain the variance associated to component number two (D). EL, ejaculation latency; EN, cumulative ejaculation number; IL, intromission latency, IN, cumulative intromission number; ML, mount latency; MN, cumulative mount number. [file Image_5.tif]
